# Patterns of foraging activity and fidelity in a southeast Asian flying fox

**DOI:** 10.1186/s40462-020-00232-8

**Published:** 2020-11-10

**Authors:** Elodie Schloesing, Rémi Chambon, Annelise Tran, Kinley Choden, Sébastien Ravon, Jonathan H. Epstein, Thavry Hoem, Neil Furey, Morgane Labadie, Mathieu Bourgarel, Hélène M. De Nys, Alexandre Caron, Julien Cappelle

**Affiliations:** 1 UMR ASTRE, CIRAD, INRAE, Université de Montpellier, Montpellier, France; 2 Université de Rennes – unité BOREA (MNHN Sorbonne Université, CNRS, UCN, IRD UA), Rennes, France; 3 UMR TETIS, CIRAD, CNRS, INRAE, AgroParisTech, Université de Montpellier, Montpellier, France; 4 Institut Pasteur du Cambodge, Phnom Penh, Cambodia; 5 EcoHealth Alliance, New York, NY USA; 6 Fauna & Flora International (Cambodia), Phnom Penh, Cambodia; 7 Harrison Institute, Sevenoaks, UK; 8 UMR ASTRE, CIRAD, RP-PCP, Harare, Zimbabwe; 9 Faculdade de Veterinaria, Universidade Eduardo Mondlane, Maputo, Mozambique; 10 UMR EPIA, Université Clermont Auvergne, INRAE, VetAgro Sup, Saint-Genès-Champanelle, France

**Keywords:** GPS telemetry, Hidden Markov models, Movement ecology, *Pteropus*, Nipah virus, Cambodia

## Abstract

**Background:**

Improved understanding of the foraging ecology of bats in the face of ongoing habitat loss and modification worldwide is essential to their conservation and maintaining the substantial ecosystem services they provide. It is also fundamental to assessing potential transmission risks of zoonotic pathogens in human-wildlife interfaces. We evaluated the influence of environmental and behavioral variables on the foraging patterns of *Pteropus lylei* (a reservoir of Nipah virus) in a heterogeneous landscape in Cambodia.

**Methods:**

We employed an approach based on animal-movement modeling, which comprised a path-segmentation method (hidden Markov model) to identify individual foraging-behavior sequences in GPS data generated by eight *P. lylei*. We characterized foraging localities, foraging activity, and probability of returning to a given foraging locality over consecutive nights. Generalized linear mixed models were also applied to assess the influence of several variables including proxies for energetic costs and quality of foraging areas.

**Results:**

Bats performed few foraging bouts (area-restricted searches) during a given night, mainly in residential areas, and the duration of these decreased during the night. The probability of a bat revisiting a given foraging area within 48 h varied according to the duration previously spent there, its distance to the roost site, and the corresponding habitat type. We interpret these fine-scale patterns in relation to global habitat quality (including food-resource quality and predictability), habitat-familiarity and experience of each individual.

**Conclusions:**

Our study provides evidence that heterogeneous human-made environments may promote complex patterns of foraging-behavior and short-term re-visitation in fruit bat species that occur in such landscapes. This highlights the need for similarly detailed studies to understand the processes that maintain biodiversity in these environments and assess the potential for pathogen transmission in human-wildlife interfaces.

## Background

Foraging can be viewed as one of the most fundamental activities of wild animals [1] because it contributes significantly to maintaining physiological functions and individual fitness [2–4]. Foraging patterns vary widely at inter- and intra-specific levels [5–7] and can be characterized by differences in foraging localities and fidelity [8, 9], as well as timing, duration and rate of foraging bouts [10, 11]. More generally, they are typically assumed to be a function of specific physiological needs, intrinsic individual features and particular environmental conditions.

Habitat quality and more specifically food-resource characteristics (i.e. diversity, abundance, nutritional value, and availability dynamics) are known to influence patterns of foraging behavior [12, 13]. For instance, animals may concentrate and increase their foraging effort in habitats where high-quality resources are available [14, 15]. Foraging animals may also revisit highly-profitable areas more often when food availability is predictable over time [16, 17]. As a consequence, habitat transformation and landscape heterogeneity can influence their foraging behavior substantially [18, 19], by forcing or allowing them to adjust their strategies which in some cases can result in local extinction or population growth [20, 21]. Because human activities are a major driver of rapid habitat modification [22], research on the foraging ecology of species at human-wildlife interfaces is crucial to understanding foraging patterns and predicting their evolution in a conservation framework [23–25].

Bats are highly represented among mammals [26] and provide ecologically important and economically significant services which include insect-pest control, plant pollination, and seed dispersal [27–29]. Almost half of all living bat species are threatened by substantial population declines or extinction [26]. A major cause of this is loss and degradation of roost and foraging resources [30, 31] due to widespread transformation of natural habitats (e.g., agricultural expansion and urbanization). As a result, improved understanding of the foraging ecology of bats in the face of ongoing habitat transformation is important for effective bat conservation. Further, because some bat species are hosts for important zoonotic viruses [32] such as lyssaviruses, filoviruses, coronaviruses and henipaviruses (Hendra and Nipah viruses) which can be transferred through contact with bat bodily fluids (e.g., saliva, urine and feces) [33], knowledge of bat foraging ecology is also essential to assessing potential risks of zoonotic transmission at human/bat interfaces. This presents challenges because bats are primarily nocturnal and highly mobile animals (i.e. powered-flight [34]) which cannot be tracked by unaided human vision. This has historically rendered monitoring of individual behavior difficult, although recent advances in satellite telemetry have partly overcome these challenges, in enabling data collection on individual locations over time [35].

We investigated the foraging ecology of Lyle’s flying fox (*Pteropus lylei*), a bat species belonging to the *Pteropus* genus which is widely distributed in Southeast Asia [36]. *Pteropus* bats face huge habitat transformation across their range due to large-scale deforestation, mainly for agriculture and, to a lesser extent, increased urbanization [30, 31]. They have also been identified as a reservoir for Nipah viruses that induce severe encephalitis in humans with high fatality rates [37, 38]. The ecology of most *Pteropus* species is relatively poorly known, although some taxa have evidently adapted to anthropogenic environments. For instance, some species populations mainly forage in human-shaped landscapes [39] and include cultivated or exotic plant species in their diet [40, 41].

We employed GPS (Gobal Positioning System) devices to collect spatio-temporal data on individual *P. lylei* and investigate their foraging ecology in a highly anthropogenic and heterogeneous environment in Cambodia. Based on GPS locations (presence data), random locations (pseudo-absence data) and environmental factors, a previous study investigated habitat selection in the same population to predict its overall distribution in the study region [42]. This found that residential areas were the preferred foraging habitat for the species, followed by plantations and unmanaged tree vegetation. In addition, *P. lylei* bats have been shown to forage in several areas each night, undertaking small-scale movements in each foraging area and frequently returning to certain areas [42, 43]. However, these individual behaviors have yet to be studied in depth to characterize the foraging behavior of the species and understand the resulting distribution patterns. More specifically, analyzing animal movements at the path-level should enhance knowledge into proximal mechanisms of animals space use [44, 45]. To this end, we employed an approach based on animal-movement modeling, which comprised a path-segmentation method (hidden Markov model) to identify individual foraging-behavior sequences from GPS data. We characterized foraging localities, foraging activity within nights, and the probability of a bat revisiting a given foraging locality over consecutive nights. We also assessed the influence of several variables on these behaviors such as proxies for energetic costs and quality of foraging areas.

## Methods

### Study region and population

The study was conducted in 2016 within the Koh Thom District of Kandal Province in Cambodia. The landscape of the region is characterized by habitats which include agricultural lands (mainly wet rice fields), fruit plantations (mainly sapodilla *Manilkara zapota* and mango *Mangifera indica*), tree vegetation (e.g., tree groves, flooded vegetation and scrublands), and residential areas (e.g., houses and backyards with a high diversity of fruit trees, including mango, bananas (*Musa spp*.), sapodilla, longan (*Dimocarpus longan*), palms (Arecaceae), kapok (*Ceiba pentandra*), guava (*Psidium guajava*) and figs (*Ficus spp.*) [42]). The studied population of Lyle’s flying foxes roosted in a grove of 21 trees located in the ‘Wat Pi Chey Saa Kor’ Buddhist pagoda (11.200 N, 105.058 E) of the Kom Poung Kor village. Between 4000 to 7000 individuals typically roost at the site during the day (but some of which occasionally roost at nearby sites), and consume fruits obtained from trees within (or on the boundaries of) the various habitats comprising the landscape [42, 46, 47].

### GPS-data collection

We caught *P. lylei* during their annual birthing period at the roost site, from 18th April to 6th May 2016 (which encompassed late pregnancy and early lactation). This period corresponds with high Nipah-virus circulation in *P. lylei* in Cambodia [47] and Thailand [48]. Selected individuals (adults only, excluding reproductively active females) were temporarily equipped with GPS devices (model: FLR V, Telemetry Solution™; spatial accuracy: 5 m; weight: 20 g) which were attached to their necks with nylon collars. The GPS loggers represented on average 4.2% ± 0.6 SD (range: 3.4–4.8%) of individual body mass (486.1 g ± 66.5, range, 414–590 g). Details on the timing of captures, anesthesia, sex, body mass, collar attachment and number of bats caught are given by [42].

Spatial data from the GPS loggers were collected for several consecutive nights (18:00–05:00) for each bat and were retrieved at the roost site in daytime using a receiving station with a maximum transfer distance of 20 m (these data are available in movebank.org; study name: “Foraging movements of Lyle’s flying foxes in Cambodia”: [42]). Acquisition rates for spatial data were heterogeneous among individuals [42]. Because [49] found that *Pteropus spp*. spent an average 12.0 min (range: 1–67 min) and 25.0 min (range: 10–40 min) searching for and consuming fruits in fig and mango trees respectively, only GPS devices recording one location every 5 min were considered. The present analyses involved seven males and one female for which data were obtained for 3 to 12 consecutive nights from 22nd April and 17th May (one additional bat was excluded due to insufficient data). The number of nights for which data were collected from a given GPS device depended on several factors including battery life and proximity of bats to the receiving station. The first night of data was excluded from analyses to avoid potential behavioral biases related to the capture event and because the GPS devices were programmed to record at a lower rate on the first night [42]. Seven nights that had more than 10% of values missing and five nights where the intervals between two locations were greater than 5 min were also excluded. For the remainder of the dataset, occasional missing values (< 2% of all data) were interpolated using continuous-time correlated random walk modelling ([50]; ‘crawl’ R package) to maintain temporal regularity between locations. The nightly movements of bats are presented in the supplementary material (Fig. S1).

### Behavioral-state identification

The nightly foraging activity of *Pteropus* species includes several types of behavior [39, 43]: *(i)* long-distance movements (hereafter referred to as commuting flights, CFs) between roost site and foraging areas, and between distinct foraging areas, *(ii)* short-distance movements between patches of high resource density within a given foraging area to collect food (foraging flights, FFs), and *(iii)* stationary bouts in a given foraging patch for fruit consumption or resting (stationary bouts, SBs). All of our GPS locations were recorded during one of these three types and the corresponding behavioral state (i.e. commuting flight, foraging flight, or stationary bout) was attributed to each location using a hidden Markov model (HMM; using the ‘moveHMM’ R package). This modelling approach allowed us to determine the composition of behavioral states in temporal sequences of spatial data, based on the distribution of step lengths and turning angles between consecutive locations ([44, 51]; see also studies in echolocating bats: [52, 53]). For this purpose, parameters (means) characterizing the distribution of movement metrics were specified in the modelling procedure for each behavioral state as follows (for step lengths and turning angles, respectively): 2000 m and 0° for commuting flights, 400 m and 0° for foraging flights, and 30 m and 180° for stationary bouts. These values were estimated from the distribution histograms of total step lengths and turning angles (see supplementary material, Fig. S2), and were consistent with our knowledge of *Pteropus* movements (e.g., low-speed and more circular movements for foraging flights and stationary bouts in comparison to commuting flights: [42, 43]). Following [51], the model was fitted with a gamma step length distribution and a von Mises turning angle distribution. As the model estimates the probability of each behavioral-state (commuting flights, foraging flights or stationary bouts) for individual locations, the state with the greatest probability was adopted for each location (values for state with the highest probabilities ranged from 0.44 to 1).

### Foraging-area characterization

**Fig. 1:**
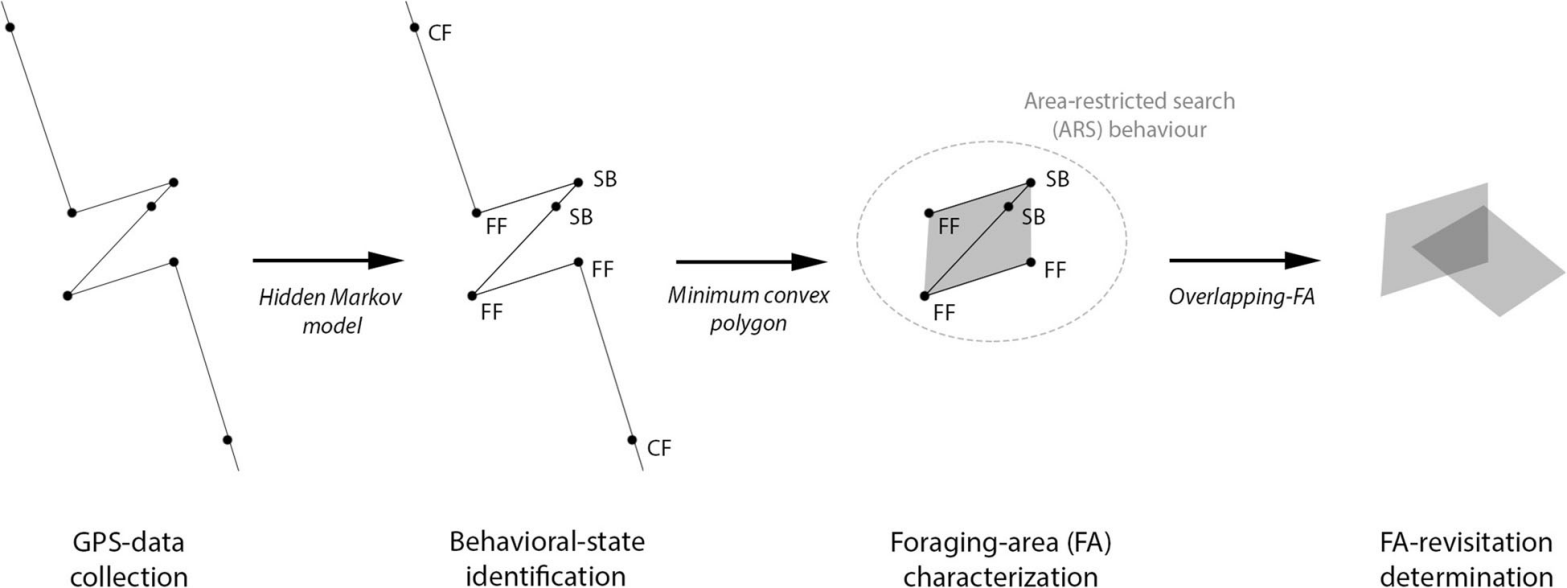
Main data-processing steps performed in analyses: *(i)* acquisition of spatio-temporal data using GPS devices attached to bats, *(ii)* identification and attribution of behavioral states (CF: commuting flights between distant areas; FF: foraging flights within a given area to collect food; SB: stationary bouts within a foraging patch to consume food collected or for resting) for each location recorded using a hidden Markov model (HMM), *(iii)* collation of all consecutive foraging activities (FF and SB) in a unique behavior (ARS: area-restricted search) to identify and characterize foraging areas (FAs), notably by generating minimum convex polygons (grey polygons) for these, and *(iv)* determination of FAs revisited over time through identification of overlapping FAs belonging to different temporal sequences of behavior undertaken by each bat. In this example, the sequence of locations (and corresponding behaviors) begins at the highest point and ends at the lowest point. Further details are given in the Methods

We employed a foraging behavior termed “area-restricted search” (ARS; also employed by [54–56]) to characterize the foraging areas used by the bats (zones where they actively search for and consume fruit, then potentially rest; FAs). In the temporal sequences of behavioral states, one ARS behavior was defined as a sequence of foraging flight and stationary bout behavioral states in variable order (e.g., FF-SB-SB-FF-FF; see Fig. 1) or as a single foraging flight or stationary bout when no sequence occurred; two ARS behaviors being separated by at least one commuting flights.

As a consequence, each ARS behavior implied one FA for which the habitat type was determined (i.e. agricultural land, plantation, tree vegetation, or residential area) using available imagery for each location in Google Earth ver. 7.1 (image resolution was sufficient to achieve this for every location [42]). When different habitat types existed within a given FA (13.2% of FAs), the habitat most represented in terms of the number of locations was selected. Minimum convex polygons (MCPs) were created for each FA in QGIS ver. 3.4.1 (Fig. 1), although this was not possible for FAs where less than three locations were recorded (21% of FAs). In addition, the distance (Euclidean, in km) of each FA to the roost site (starting location of a bat on a given night) was calculated by averaging the distance of all FA locations to this site. The duration spent by a bat within a given FA was calculated (in minutes) using the time elapsed between all consecutive locations constituting the ARS behavior.

Finally, we identified FAs that were used several times by a given bat during the study (hereafter referred to as “re-visitation” behavior). To this end, we computed the mean distance between all pairs of locations constituting each minimum convex polygon (MCP; range: 1.3–530.3 m depending on the MCP). The median (37.0 m) of these averaged distances was used to create a buffer (radius: 18.5 m) around each MCP for FAs including at least three locations and around each location for FAs including less than three locations. These buffers were then used to identify overlapping FAs and thus to determine FAs that were revisited (Fig. 1).

### Statistical analyses

We characterized foraging localities, nightly foraging activity and foraging-area re-visitation for bats. Overall, the effect of habitat type was tested as a proxy for the global quality of habitats used for foraging, mainly in terms of their food resources (i.e. typical diversity, abundance, renewal rate, availability, and energy content). In this context, the food-resource quality of residential areas was regarded as particularly high given the diversity (see the main species previously cited in the Methods - Study region and population; also see [47]) and abundance of fruit trees (with at least one tree in 93.5% of households, and up to 200 of the same species in a single backyard: [47]), plus the fact that fruits were frequently unharvested [47]. However, sample sizes were relatively low for agricultural lands (only 17 ARS behaviors occurred in this habitat type) compared to other habitats (74, 84, 159 ARS behaviors in tree vegetation, plantations and residential areas, respectively). As such, for statistical issues, agricultural lands were discarded from our analyses of the effect of the habitat type (details of these analyses and the number of ARS behaviors taken into account are given below).

#### Foraging localities

We tested whether the number of ARS behaviors displayed by a bat during a night differed between habitat types (*n* = 317 ARS behaviors). For this purpose, we used a Generalized Linear Mixed Model (GLMM; Poisson error distribution and log link function) which included habitat type as a fixed effect and the identity of individuals as an additive random effect to account for repeated measurements (several nights per individual; random effect also used in all subsequent mixed models). In addition, we used a GLMM (Gamma error distribution and log link function) to test whether the distance between the FA associated with each ARS behavior (*n* = 317 ARS behaviors) and the roost site was dependent on the habitat type of the FA (explanatory variable).

#### Nightly foraging activity

We compared several GLMMs (Poisson error distribution and log link function) with different combinations of explanatory variables to investigate variation in the number of ARS behaviors displayed by a bat at night (*n* = 334 ARS behaviors). Candidate explanatory variables included the total duration devoted to commuting flights on a given night (as a proxy for the energy costs related to overall nightly activity), and the total duration of ARS behavior in residential areas per night (as a proxy for the degree of utilization of a supposed high-quality habitat type). Following this, we explored variables influencing the duration of ARS behavior (*n* = 317 ARS behaviors) by comparing several GLMMs (Gamma error distribution and log link function). More specifically, we tested whether habitat type influenced this duration and we also tested the influence of the total ARS-behavior duration prior to the ARS behavior considered, since the beginning of the night (as a proxy for the degree their energetic needs had been satisfied).

#### Short-term fidelity to foraging areas

We finally compared several GLMMs (Binomial error distribution and logit link function) to investigate variation in the probability of a bat revisiting a given FA at least once in the following 48 h (two nights). Because Nipah virus typically survives in the environment for a few hours [57] and as long as a few days under optimal conditions, this period was of specific interest in terms of local pathogen-accumulation and transmission risks (*n* = 179 ARS behaviors). The influence of the duration of ARS-behaviors within a FA on a given night (the accumulated duration of ARS-behaviors in the rare case where a bat visited several times this FA during the same night) was tested on that probability, as a proxy for the FA quality. In addition, we tested the effect of habitat type as a proxy for a more global and integrative food-resource quality related more to habitat type than the specific FA used. We also tested the influence of the distance between the FA and the roost site (notably as a proxy for the energy costs required to reach this area).

#### Modelling procedures

All statistical analyses were carried out using R version 3.5.1 (R Core Team). GLMMs were fitted with the ‘lme4’ package. Type-II tests were performed to assess the significance of analyses evaluating the effect of a unique variable (‘car’ package). Where the influence of several candidate variables was investigated, all models containing one simple effect were computed. Models with additive effects were also computed, but interaction effects were not tested as meaningful hypotheses were lacking for these. We also computed a null (intercept-only) model. Candidate models were compared using the Akaike information criterion corrected for small sample sizes (AICc: [58]; ‘AICcmodavg’ package). A model was considered more competitive when its AICc was at least 2 units lower (ΔAICc) than others. Otherwise, we considered the AICc weight (ωAICc) as a measure of the probability of a model being the best model [58], and thus retained the model with the highest AICc weight. Estimated marginal means (EMMs, ± SE) were used to provide adjusted estimates of the response variable (‘emmeans’ package) by taking the simple effect of the other variable into account (in the case of additive effects), and also to perform post-hoc comparisons (Tukey’s comparisons). Overall, the variance explained by the effects retained was considered for each GLMM by computing the marginal and conditional R^2^ (see [59]; ‘MuMIn’ package). Quantitative explanatory variables were centered and scaled. Data on the number of ARS and FA re-visitations performed by each bat are provided in the supplementary material (Table S1 and S2).

## Results

### Foraging localities

**Fig. 2:**
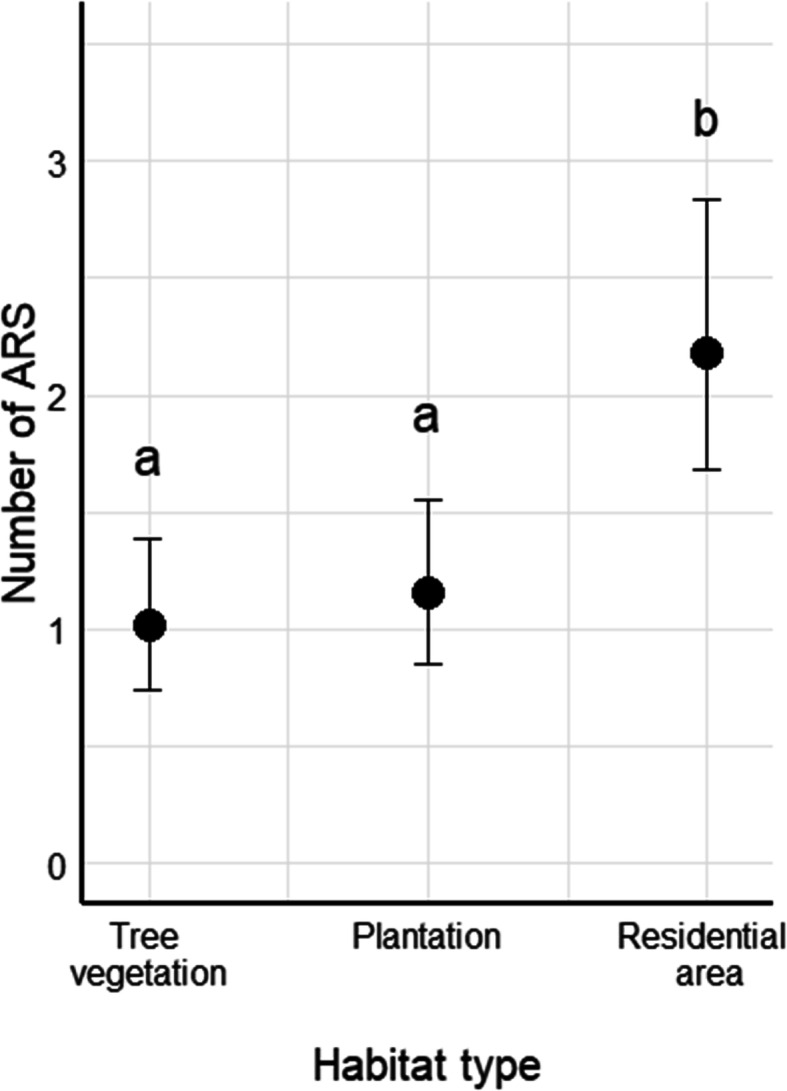
Estimation (with 95% CIs) of the number of area-restricted search (ARS) behaviors performed by a bat on a given night, according to habitat type (i.e. fruit plantations, tree vegetation, or residential areas). Significant post-hoc differences are represented by different letters (Tukey’s comparisons)

**Fig. 3:**
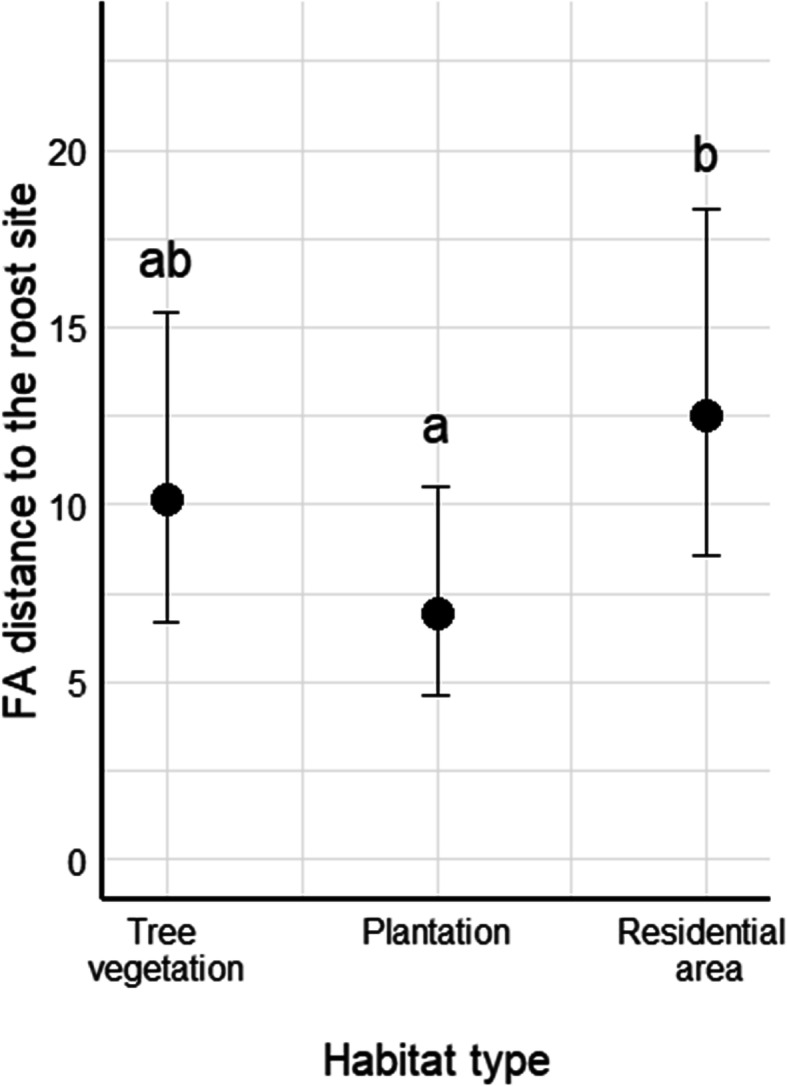
Estimation (with 95% CIs) of the distance (in km) between a foraging area (FA) and the roost site, according to the habitat type of the FA (i.e. fruit plantations, tree vegetation, or residential areas). Significant post-hoc differences are represented by different letters (Tukey’s comparisons)

The number of ARS behaviors displayed by a bat during the night differed between habitat types (χ^2^ = 39.5, df = 2, *p* < 0.001, marginal R^2^ = 0.13, conditional R^2^ = 0.23; Fig. 2): it was significantly higher in residential areas (2.2 ± 0.29) compared to other habitats (tree vegetation: 1.0 ± 0.16; fruit plantations: 1.2 ± 0.18). The distance between the foraging area associated with each ARS behavior and the roost site was dependent on the habitat type of the foraging area (χ^2^ = 15.8, df = 2, p < 0.001, marginal R^2^ = 0.07, conditional R^2^ = 0.25; Fig. 3): this distance was significantly higher for the residential areas (12.5 ± 2.4) compared to fruit plantations (7.0 ± 1.5), whereas it was intermediate for tree vegetation (10.1 ± 2.2).

### Nightly foraging activity

**Fig. 4:**
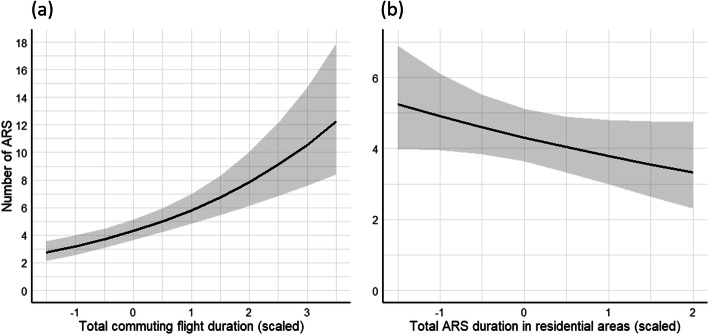
Variation (with 95% CIs) in the number of area-restricted search (ARS) behaviors exhibited by a bat during a given night, in relation to (**a**) the total duration spent in commuting flight on a given night (scaled; real values ranging from 25 to 340 min), and (**b**) the total duration of ARS behaviors in residential areas during a given night (scaled; real values ranging from 0 to 535 min)

**Fig. 5:**
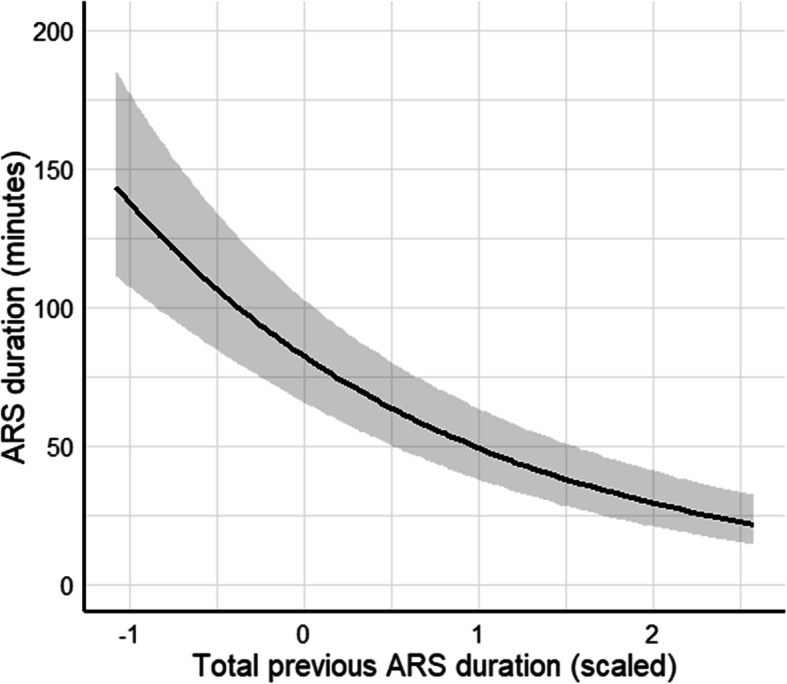
Variation (with 95% CIs) in the duration of an area-restricted search (ARS) behavior exhibited by a bat during a night, according to the total duration of ARS-behavior previously displayed by the bat since the beginning of the night (scaled; real values ranging from 0 to 575 min)

The number of ARS behaviors displayed by a bat increased significantly with the total duration spent in commuting flights and decreased with the total duration spent in residential areas (marginal R^2^ = 0.32, conditional R^2^ = 0.41; Table 1; Fig. 4a and b). The durations of ARS behavior significantly decreased with the total ARS duration spent before the ARS evaluated (marginal R^2^ = 0.19, conditional R^2^ = 0.25; Table 1; Fig. 5).

**Table 1:** General linear mixed models employed to explain variation in the number of area-restricted searches (ARS) performed by a bat during a given night, the duration of ARS behaviors, and the probability of a bat revisiting a given foraging area (FA) in the following 48 h

Response variable	Explanatory variable	AICc	ΔAICc	ωAICc
Number of ARS				
	**CF duration + Residential-area duration**	**298.60**	**0.00**	**0.54**
	CF duration	298.96	0.36	0.46
	Residential-area duration	321.98	23.38	0.00
	Null model	323.93	25.33	0.00
ARS duration				
	**Total previous duration**	**3440.59**	**0.00**	**0.64**
	Total previous duration + Habitat	3441.79	1.19	0.46
	Habitat	3487.34	46.75	0.00
	Null model	3498.68	58.08	0.0
FA-revisitation probability				
	**ARS duration + Roost-site distance + Habitat**	**161.41**	**0.00**	**0.90**
	ARS duration + Roost-site distance	166.26	4.85	0.08
	ARS duration + Habitat	168.87	7.46	0.02
	ARS duration	174.29	12.88	0.00
	Roost-site distance + Habitat	210.41	48.99	0.00
	Habitat	218.73	57.32	0.00
	Roost-site distance	225.91	64.50	0.00
	Null model	234.25	72.83	0.00

CF duration: the total duration spent in commuting flight on a given night. Residential-area duration: the total duration spent in residential areas on a given night. Total previous duration: the summed duration of all ARS behaviors before a given ARS behavior since the beginning of activity on that night. Habitat: three habitat types (i.e. fruit plantations, tree vegetation, or residential areas). ARS duration: the duration of ARS behaviors in a given FA. Roost-site distance: the distance between a given FA and the roost site. AICc: the Akaike information criterion corrected for small sample sizes. ΔAICc: the difference in AICc between any model and the model with the lowest AICc. ωAICc: may be considered as the probability that a given model is the best approximation (Akaike weight). The model retained is shown in bold for each response variable (additional details are given in the text)

### Short-term fidelity to foraging areas

**Fig. 6:**
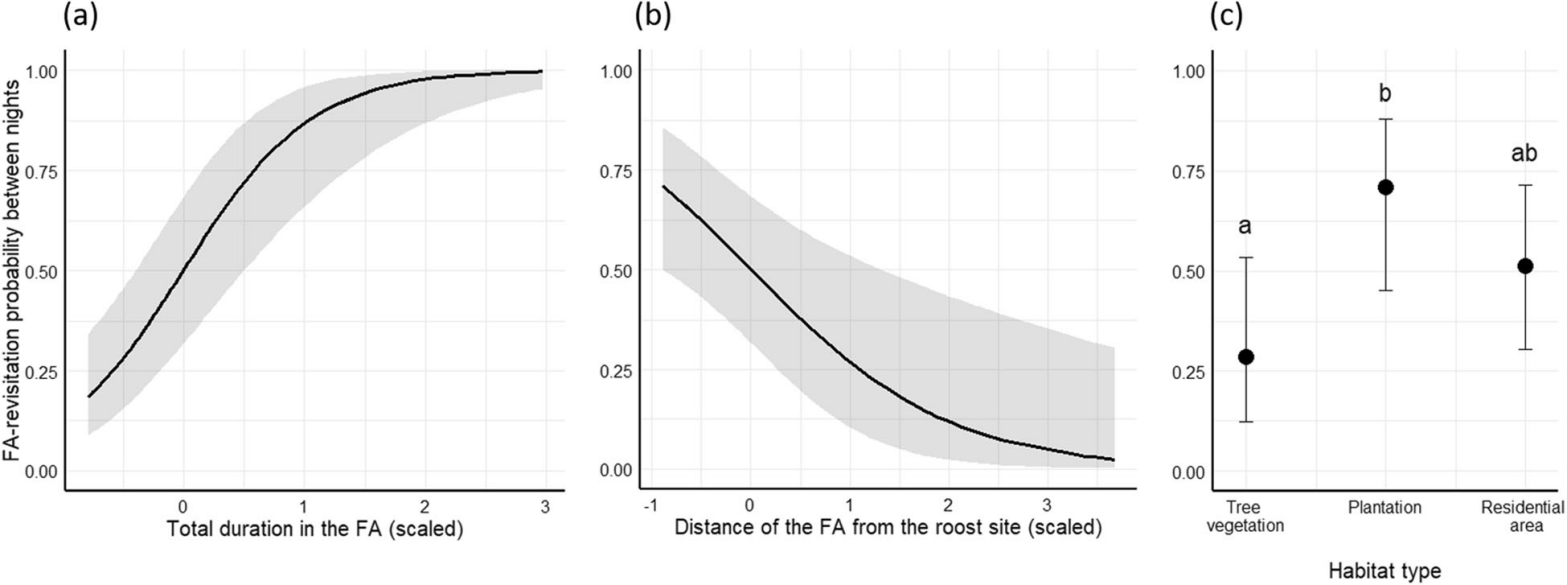
Variation (with 95% CIs) in the probability of a bat revisiting a given foraging area (FA) in the following 48 h, in relation to (**a**) the total duration of area-restricted search (ARS) behaviors displayed by the bat in the FA during the night (scaled; real values ranging from 5 to 535 min), (**b**) the distance between the FA and roost site (scaled; real values ranging from 108 to 66,901 m), and (**c**) the habitat type (i.e. fruit plantations, tree vegetation, or residential areas). Significant post-hoc differences are represented by different letters (Tukey’s comparisons)

The probability of a bat revisiting a given foraging area for two consecutive nights significantly increased with the duration previously spent in the FA, decreased with the distance between the foraging area and the roost site, and was influenced by habitat type (marginal R^2^ = 0.57, conditional R^2^ = 0.62; Table 1; Fig. 6a, b and c): this probability was significantly higher for fruit plantations (0.71 ± 0.11), lowest for tree-vegetation (0.29 ± 0.11), and intermediate for residential areas (0.51 ± 0.11).

## Discussion

### Foraging localities

During the night, a bat performed more foraging bouts in residential areas on average (Fig. 2). A previous study on the same population showed that residential areas were the preferred foraging habitat of the species, but did not detail the use of foraging localities by individual bats on a given night (e.g., number and duration of visits) [42]. In highlighting that individual bats favor residential areas by multiplying their foraging bouts in this habitat type, our work sheds further light on the distribution patterns revealed by [42]. Foraging habitats among fruit bats vary from primary forest to urban areas (e.g., in the grey-headed flying fox *Pteropus poliocephalus:* [60]; in the Indian flying fox *Pteropus giganteus*: [61]) and may change over time (in the African straw-coloured fruit bat *Eidolon helvum*: [62]; in the solitary flying fox *Pteropus dasymallus*: [63]). Habitat choice in animals is influenced by environmental constraints and food resources, which may vary seasonally [62, 63]. Given the high density of backyards in residential areas that may provide abundant and diversified food resources [64], and the documented tolerance of some bat species to anthropization [65], our results suggest that *P. lylei* is a flexible generalist and opportunistic forager during the birthing season.

The residential areas chosen by bats were more distant from their roost site compared to other habitats (Fig. 3). This may be explained in an optimal-foraging framework, whereby the energetic costs associated with increased travel distances are offset by the benefits allied with good feeding conditions [66]. In addition, our data showed that bats foraging longer in residential areas performed fewer foraging bouts during the night (Fig. 4b), and that they sometimes performed only one foraging behavior over an entire night. As the latter has been reported for other pteropodid bats in urban or semi-urban environments [67], these findings support the idea that residential areas may represent high-quality foraging localities for certain species, and that their resources may entirely support their daily energetic needs.

### Nightly foraging activity

Our results indicate that the time spent foraging by our study bats in consecutive foraging areas decreased overnight. Wild animals need information about resource availability to optimize their foraging activities [68], and due to the renewal and maturation dynamics of fruits, frugivorous species are presented with predictable food resources [69]. Consequently, we hypothesize that bats foraged for a longer period at the beginning of the night in areas with higher-quality food resources to ensure sufficient energy gains, before shifting to shorter foraging bouts to track the availability of food resources in other locations [70] and/or to complement their diets [71, 72]. Inspection of fruit resources has been reported in mangabey monkeys, which use fruiting synchrony to control specific trees and improve their foraging performance [73]. As such, the positive correlation between the number of foraging bouts and the total time spent in commuting flight over a night (Fig. 4a) may indicate that some nights were more specifically devoted to collecting information about potential foraging areas, hence supporting and further specifying our hypothesis. In addition and as discussed above, the number of foraging bouts each night was also influenced by foraging-habitat localities.

Habitat type was not retained as a predictor of foraging-behavior duration in our analyses. The marginal theorem value predicts that animals remain longer in a profitable patch [74] and is supported by several field studies on mammals [15, 75]. It is possible that residential areas represent particularly attractive foraging habitats and that a relatively important number of foraging bouts displayed in these were particularly short and used to acquire information for future nights. This might at least partly explain the lack of habitat effect on the foraging-behavior duration in our study.

### Short-term fidelity to foraging areas

Our bats revisited some foraging areas over short-term scales (in the following 48 h) according to their previous experience and environmental variables. Re-visitation behavior is widespread among animals, and may be determined by environmental heterogeneity, food-resource renewal rates and specific cognitive capabilities [76, 77]. Nonetheless, while foraging-site fidelity has been shown for many bat species [78–80], the factors influencing this behavior have received little attention. A recent study carried out on a nectar-feeding bat showed that the re-visitation of the most profitable places (i.e. in terms of quality and distance to the colony) was a consequence of a reinforcement learning strategy whereby bats avoided competition and maximized their global food intake [81].

We found the probability of a bat revisiting an area increased with the duration of the foraging behavior spent in the area (Fig. 6a). Similar positive links between short-term returns and previous visit durations (as a proxy for area quality) has also been found in other mammals and birds [82, 83]. This behavior is particularly relevant for species that do not deplete a localized food-resource in one visit or that forage in areas with high food-renewal rates (e.g., [84]), whereas longer intervals between visits may be expected in other cases (e.g., [85]). As our bats used different foraging areas, short-term returns to these may allow them to optimize their energy gains by exploiting high-quality resources in some familiar areas over consecutive nights. The negative association between re-visitation probability and distance to the roost site supports this hypothesis (Fig. 6b). Foraging areas in the vicinity of the roost site may be more familiar and visited more often (i.e. energy costs to visit them being lower). Exploration of individual GPS data (see Table S2) supports these explanations: areas revisited were relatively small in number, mainly located in the immediate vicinity of the roost site, and accumulated several visits during the study period. More generally, the influence and benefits of site familiarity on re-visitation behavior of bats has also been described for other mammals and birds during the breeding season [86–88].

In addition, foraging areas in fruit plantations were revisited more often by our bats, followed by residential areas (Fig. 6c). Habitat characteristics are known to influence re-visitation behavior in mammals [85, 89]. Even if plantations are especially attractive for plant-visiting bats [90], most commercially-grown fruits are harvested too early for flying-fox consumption [91]. Only damaged or forgotten fruits reach sufficient maturity, which limits their availability and predictability for bats. One biological interpretation for our result could be the existence of specific features in some plantations (e.g., abandoned sites and attractive fruiting trees along plantation edges), resulting in high re-visitation rates for a few familiar areas (this hypothesis is partly supported by our GPS data; see Table S2). In contrast, given the hypothesized high foraging quality of residential areas, we suggest that the wide range of fruit trees (regarding species and fruiting phenology) and associated food resources has led to a heterogeneous degree of short-term attraction between the residential areas visited. Finally, the lowest probability found for tree-vegetation habitats might be attributable to their possessing lower diversity and quantity of food resources during our study period, but we cannot evaluate this possibility as the relevant information is currently lacking. Overall, our results suggest that the reinforcement learning strategy previously mentioned [81] may have led to the re-visitation pattern found in our work, but quantitative information about food quality and quantity in each foraging locality are needed to support this hypothesis.

### Limitations and future prospects

Contrasting degrees of inter-individual variability regarding the behavior studied is suggested by individual data exploration (see Table S1 and S2), explained variance (both marginal and conditional R^2^) and confidence intervals for all results. Inter-individual variation in foraging could result from differences in personality traits, previous experience of individuals (e.g., familiarity with food distribution and predictability) and degrees of specialization [92]. In association with the solitary nature of foraging fruit bats [78], we suggest that the heterogeneity of our study region (e.g., habitat types, food-resource diversity and availability) promoted different individual foraging strategies. Nevertheless, our small sample size and limited sampling period preclude conclusions regarding the prevalence of these behaviors in the wider population and their seasonal consistency. Investigation of other intrinsic variables (e.g., age, sex and body condition) and long-term monitoring of individual bats (using Argos telemetry) would likely provide some interesting perspectives.

Our study provides new insights into the foraging ecology of *Pteropus lylei* whose patterns of re-visitation behavior appear to be relatively complex. These patterns were significant but influenced by several variables and were likely constrained to relatively few areas at individual levels. Furthermore, the prevalence of Nipah virus in this population is 0.9 and 0.2% during the pregnancy and lactation, respectively (i.e. the reproductive periods encompassed by the study) [47]. Since the return of an infected bat to a given area over consecutive nights could increase probability of pathogen accumulation there, our findings suggest that risks of transmission from infected bats could be highly localized, at least during the short term. More generally, our work emphasizes the importance of considering individual-movement patterns in epidemiological frameworks. However, the complexity of foraging patterns revealed by our study (correlates and inter-individual variation) also means investigation of transmission pathways and prevention of spillover risks will be challenging, particularly given the ongoing expansion of human-wildlife interfaces in Southeast Asia.

## Conclusions

Our study employed a path-segmentation method (hidden Markov model) to elucidate the behavioral sequences of individual *P. lylei* from GPS data. Our results suggest that environmental and individual features (e.g., availability and quality of food resources, habitat-familiarity and experience of bats) have a significant bearing on their patterns of foraging behavior and fidelity to a given foraging area over consecutive nights. As *P. lylei* is a natural reservoir for Nipah virus, this is particularly relevant to viral transmission risks and indicates that fine-scale ecological studies of species capable of adapting to newly anthropized environments will be important to understand the ecological processes that maintain biodiversity in these landscapes and assess the potential for pathogen transmission in human-wildlife interfaces.

## Supplementary Information

**Additional file 1: Fig. S1.** Foraging tracks of 8 individuals of *Pteropus lylei* equipped with GPS loggers, in Cambodia.

**Additional file 2: Fig. S2.** Distribution of step lengths and turning angles related to the GPS data.

**Additional file 3: Table S1.** Mean number (± SD) of area restricted search (ARS) behaviors displayed by each bat in relation to habitat type.

**Additional file 4: Table S2.** Number of foraging-area (FA) re-visitation behaviors displayed by each individual, number of FAs that were used several times by a bat during the study period according to the habitat type, and distance (m) range between FAs used several times and the roost site.

## Data Availability

The GPS data supporting the conclusions of this article are available on Movebank (movebank.org, study name “Foraging movements of Lyle’s flying foxes in Cambodia (data from [42])”) and are published in the Movebank Data Repository [42]. 10.5441/001/1.j25661td.

## Abbreviations

AIC: Akaike Information Criterion
ARS: Area-Restricted Search
CF: Commuting Flight
EMM: Estimated Marginal Mean
FA: Foraging Area
FF: Foraging Flight
GLMM: Generalized Linear Mixed Model
GPS: Gobal Positioning System
HMM: Hidden Markov Model
MCP: Minimum Convex Polygon
SB: Stationary Bout
