# Supplementary material for: Patterns of foraging activity and fidelity in a southeast Asian flying fox
Source: Mov Ecol. 2020 Nov 10;8:46. doi: 10.1186/s40462-020-00232-8 (PMC7652672; doi:10.1186/s40462-020-00232-8)
Supplement: Supplementary file 1 — Additional file 1: Fig. S1. Foraging tracks of 8 individuals of Pteropus lylei equipped with GPS loggers, in Cambodia. [file 40462_2020_232_MOESM1_ESM.docx]

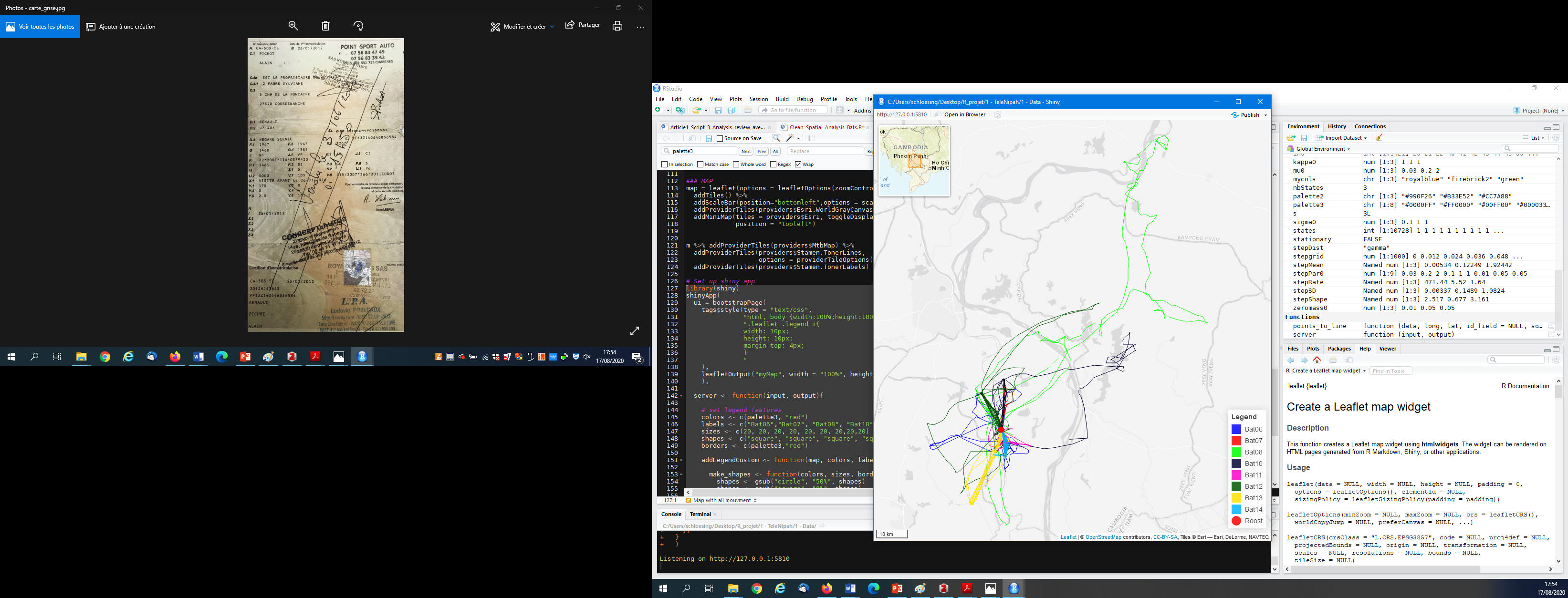


**Fig. S1.** Foraging tracks of 8 individuals of *Pteropus lylei* equipped with GPS loggers, in Cambodia. GPS devices were programmed to record one location every 5 minutes over several nights (see the Methods for more details). For each individual (see the legend of the different colors on the figure) and night, the departure site and the last location was the roost site (red point).
