# Supplementary material for: Patterns of foraging activity and fidelity in a southeast Asian flying fox
Source: Mov Ecol. 2020 Nov 10;8:46. doi: 10.1186/s40462-020-00232-8 (PMC7652672; doi:10.1186/s40462-020-00232-8)
Supplement: Supplementary file 2 — Additional file 2: Fig. S2. Distribution of step lengths and turning angles related to the GPS data. [file 40462_2020_232_MOESM2_ESM.docx]

(a)


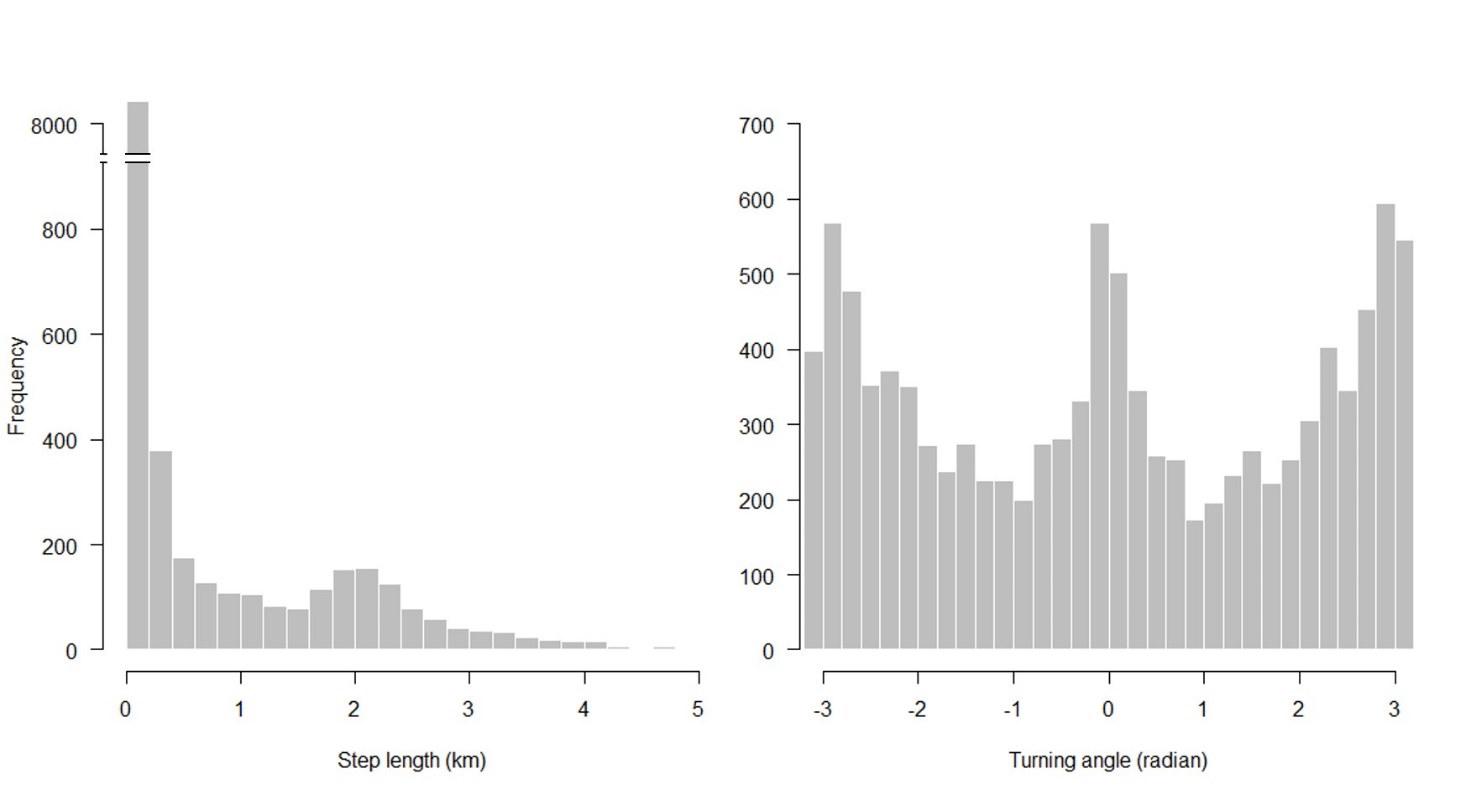


(b)


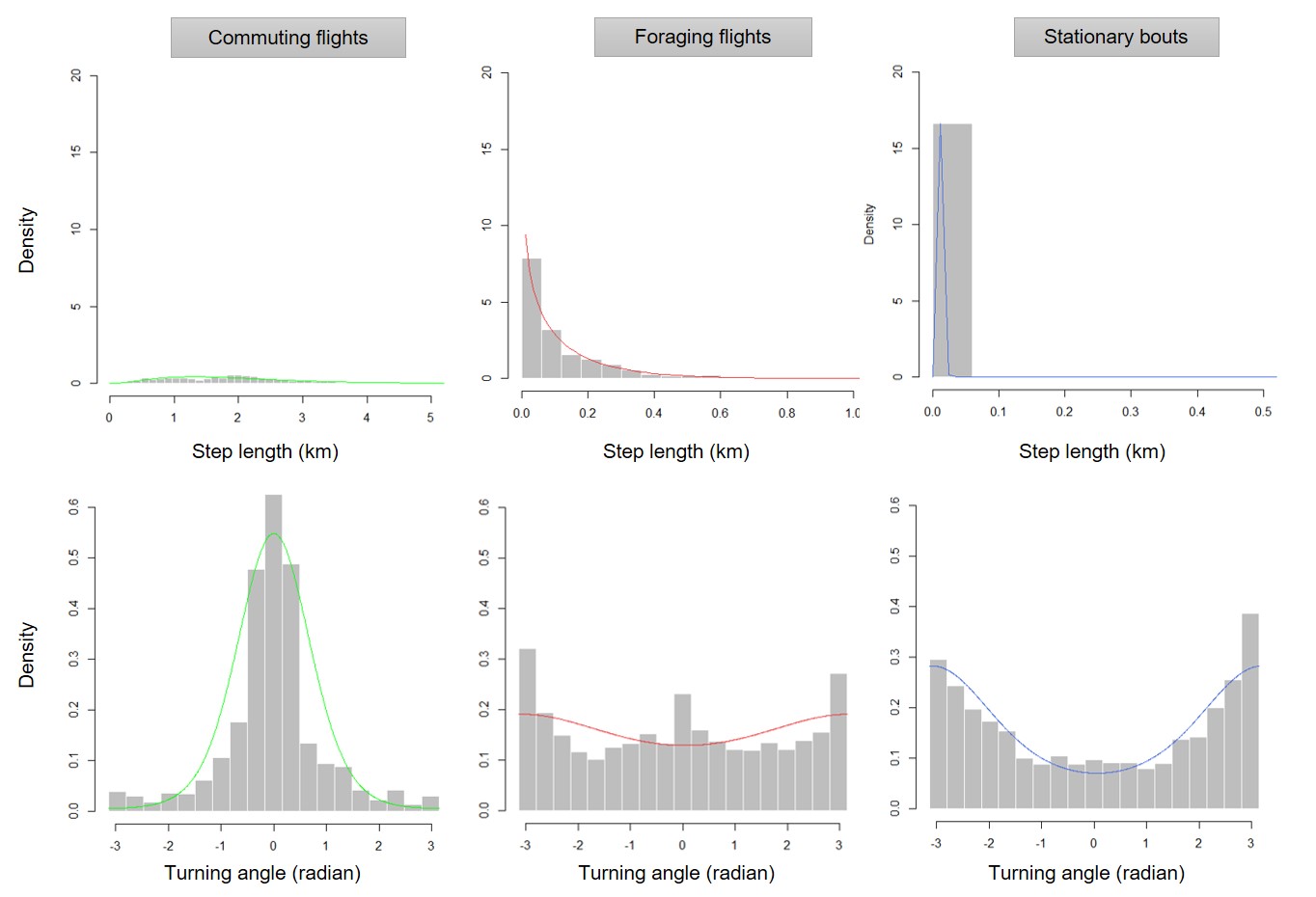


**Fig. S2**. Distribution of step lengths and turning angles related to the GPS data. (a) Overall distributions. The hidden Markov model was fitted by specifying the parameters (means) characterizing the typical distribution of step length and turning angle of each behavioral state. Three behavioral states were expected for *P. lylei* (see the Methods for more details). Parameters for commuting flights (2000 m which corresponds to a peak on the histogram and 0° (0 rad) which corresponds to a flight in straight line), foraging flights (400 m which corresponds to an inflection point on the histogram and 0° (0 rad) which corresponds to a flight in straight line) and stationary bouts (30 m which corresponds to numerous short values on the histogram and 180° (π rad) which corresponds to circular movements) were visually estimated from these two histograms. (b) Distributions among the three behavioral states (stationary bouts, foraging flights and commuting flights) derived from the hidden Markov model.
