# Supplementary material for: Patterns of foraging activity and fidelity in a southeast Asian flying fox
Source: Mov Ecol. 2020 Nov 10;8:46. doi: 10.1186/s40462-020-00232-8 (PMC7652672; doi:10.1186/s40462-020-00232-8)
Supplement: Supplementary file 3 — Additional file 3: Table S1. Mean number (± SD) of area restricted search (ARS) behaviors displayed by each bat in relation to habitat type. [file 40462_2020_232_MOESM3_ESM.docx]

**Table S1.** Mean number (± SD) of area restricted search (ARS) behaviors displayed by each bat in relation to habitat type.

| Bat ID |  | Number of nights |  | Number of ARS behaviors displayed by a bat during the night | | | | | |  |
| --- | --- | --- | --- | --- | --- | --- | --- | --- | --- | --- |
|  |  |  |  | Tree vegetation | Plantation | Residential area | Agricultural land | Total including agricultural land | Total excluding agricultural land | |
| Bat06 |  | 11 |  | 2.3 ± 1.0 | 1.6 ± 0.8 | 2.5 ± 1.6 | 0.5 ± 0.7 | 6.9 ± 0.1 | 6.4 ± 0.1 |  |
| Bat07 |  | 3 |  | 2.0 ± 2.0 | 0.0 ± 0.0 | 2.0 ± 1.0 | 0.3 ± 0.6 | 4.3 ± 0.5 | 4.0 ± 0.5 |  |
| Bat08 |  | 8 |  | 0.6 ± 0.7 | 1.0 ± 0.8 | 4.8 ± 2.0 | 0.2 ± 0.5 | 6.6 ± 0.3 | 6.4 ± 0.3 |  |
| Bat10 |  | 6 |  | 1.8 ± 1.0 | 1.8 ± 0.8 | 1.5 ± 1.4 | 0.3 ± 0.8 | 5.5 ± 0.2 | 5.2 ± 0.2 |  |
| Bat11 |  | 11 |  | 1.4 ± 1.4 | 2.0 ± 1.3 | 0.6 ± 0.7 | 0.0 ± 0.0 | 4.0 ± 0.1 | 4.0 ± 0.1 |  |
| Bat12 |  | 11 |  | 0.6 ± 0.8 | 0.7 ± 0.8 | 3.1 ± 2.1 | 0.5 ± 0.5 | 4.9 ± 0.1 | 4.5 ± 0.2 |  |
| Bat13 |  | 9 |  | 0.1 ± 0.3 | 1.4 ± 0.9 | 2.4 ± 1.3 | 0.1 ± 0.3 | 4.1 ± 0.1 | 4.0 ± 0.1 |  |
| Bat14 |  | 12 |  | 0.3 ± 0.7 | 0.3 ± 0.7 | 1.3 ± 0.5 | 0.0 ± 0.0 | 1.9 ± 0.1 | 2.0 ± 0.1 |  |

The identity of the bat (Bat ID) is consistent with the work of Choden et al. (2019). Number of nights: number of nights over which GPS data were considered for each bat in the study. For each bat, the mean (± SD) total number of ARS performed during the night is given when considering agricultural lands or not (see the main text).
