# Supplementary material for: Patterns of foraging activity and fidelity in a southeast Asian flying fox
Source: Mov Ecol. 2020 Nov 10;8:46. doi: 10.1186/s40462-020-00232-8 (PMC7652672; doi:10.1186/s40462-020-00232-8)
Supplement: Supplementary file 4 — Additional file 4: Table S2. Number of foraging-area (FA) re-visitation behaviors displayed by each individual, number of FAs that were used several times by a bat during the study period according to the habitat type, and distance (m) range between FAs used several times and the roost site. [file 40462_2020_232_MOESM4_ESM.docx]

**Table S2.** Number of foraging-area (FA) re-visitation behaviors displayed by each individual, number of FAs that were used several times by a bat during the study period according to the habitat type, and distance (m) range between FAs used several times and the roost site.

| Bat ID | Number  of nights | Number of FA-revisitation  behaviors | Number of FAs used several times | | | | Distance range between FAs used several times and the roost site (m) |
| --- | --- | --- | --- | --- | --- | --- | --- |
|  |  |  | Tree vegetation | Plantation | Residential area | Total |  |
| Bat06 | 8 | 23 | 4 (6) | 2 (9) | 3 (8) | 9 | 472 – 10 972 |
| Bat07 | 1 | 1 | 0 | 0 | 1 (1) | 1 | 7637 |
| Bat08 | 6 | 9 | 0 | 1 (3) | 4 (6) | 5 | 109 – 42 235 |
| Bat10 | 2 | 2 | 0 | 1 (2) | 0 | 1 | 20 343 |
| Bat11 | 9 | 15 | 2 (6) | 1 (9) | 0 | 3 | 4601 – 6373 |
| Bat12 | 9 | 9 | 0 | 0 | 2 (9) | 2 | 345 – 13 552 |
| Bat13 | 5 | 14 | 0 | 2 (7) | 2 (7) | 4 | 34 – 24 721 |
| Bat14 | 10 | 11 | 0 | 1 (1) | 1 (10) | 2 | 4817 – 7827 |

The identity of the bat (Bat ID) is consistent with the work of Choden et al. (2019). Number of nights: number of nights over which FA-revisitation behavior was assessed for each bat. Numbers in brackets represent the number of FA-revisitation behaviors performed by each bat according to the habitat type. Distance range between FAs used several times and the roost site corresponds to the minimal and maximal values. All distances between FAs and the roost site considered in the whole study ranged from 34 to 66 901 m.
